# Transcriptome Remodeling of Differentiated Cells during Chronological Ageing of Yeast Colonies: New Insights into Metabolic Differentiation

**DOI:** 10.1155/2018/4932905

**Published:** 2018-01-11

**Authors:** Derek Wilkinson, Jana Maršíková, Otakar Hlaváček, Gregor D. Gilfillan, Eva Ježková, Ragnhild Aaløkken, Libuše Váchová, Zdena Palková

**Affiliations:** ^1^Faculty of Science, Charles University, BIOCEV, 252 50 Vestec, Czech Republic; ^2^Institute of Microbiology of the Czech Academy of Sciences, BIOCEV, 252 50 Vestec, Czech Republic; ^3^Department of Medical Genetics, Oslo University Hospital and University of Oslo, 0450 Oslo, Norway

## Abstract

We present the spatiotemporal metabolic differentiation of yeast cell subpopulations from upper, lower, and margin regions of colonies of different ages, based on comprehensive transcriptomic analysis. Furthermore, the analysis was extended to include smaller cell subpopulations identified previously by microscopy within fully differentiated U and L cells of aged colonies. New data from RNA-seq provides both spatial and temporal information on cell metabolic reprogramming during colony ageing and shows that cells at marginal positions are similar to upper cells, but both these cell types are metabolically distinct from cells localized to lower colony regions. As colonies age, dramatic metabolic reprogramming occurs in cells of upper regions, while changes in margin and lower cells are less prominent. Interestingly, whereas clear expression differences were identified between two L cell subpopulations, U cells (which adopt metabolic profiles, similar to those of tumor cells) form a more homogeneous cell population. The data identified crucial metabolic reprogramming events that arise de novo during colony ageing and are linked to U and L cell colony differentiation and support a role for mitochondria in this differentiation process.

## 1. Introduction

Yeast colonies are multicellular communities of cells that organize themselves in space and have the ability to differentiate and form specialized subpopulations that fulfill specific tasks during colony development and ageing [1–5]. Despite the fact that mechanisms driving colony development and differentiation are largely unknown, indications exist that the formation of gradients of nutritive compounds such as oxygen and metabolites (including low Mw compounds and waste products) released by cells localized in different positions within the structure contributes to the formation of specialized cell subpopulations [6–8].

*Saccharomyces cerevisiae* colonies that are grown on complete respiratory medium periodically alter the pH of their surroundings, switching from an acidic phase to a period of alkalization and back. Alkali phase is accompanied by production of volatile ammonia, which functions as a signal that contributes to colony metabolic reprogramming [9–11]. Ammonia (produced by a neighboring colony or even coming from an artificial source) is able to prematurely induce ammonia production (and thus the transition to alkali phase) in acidic-phase colonies [10, 12]. Using microarray transcriptomic analysis and different biochemical and molecular biology approaches, we have previously characterized two major morphologically distinct cell subpopulations that are formed within *S. cerevisiae* colonies during the alkali developmental phase. These subpopulations are differently localized in central areas of the colonies: the U cell subpopulation forms upper-cell layers, whereas L cells form lower layers of these colonies [6, 13]. Despite the fact that U/L cell colony differentiation occurs in relatively old colonies (older than 12 days) that are composed of mostly stationary-phase cells, U cells behave as metabolically active cells, display a longevity phenotype, and exhibit specific metabolism. For example, U cells activate the TORC1 pathway, which is not typical of stationary-phase cells. These cells also display decreased mitochondrial activity compared with L cells. Several metabolic features of U cells are similar to those of cells of solid tumors [6]. In contrast, L cells, despite being localized from the beginning of colony growth close to nutritive agar, behave as starving and stressed cells that begin losing viability earlier than U cells [6]. These earlier studies showed that L cells release nutritive compounds that are consumed by U cells and are important to U cell survival and long-term viability. In addition to direct measurements of the release and consumption of amino acids and sugars by U and L cells, we showed that mutants with increased viability of L cells often have decreased viability of U cells [6, 7]. Despite prominent differences in the physiology and morphology of U and L cells, we discovered recently that L cells are not homogeneous, but include two subpopulations that differ in the specificity of mitochondrial retrograde signaling. Retrograde signaling, identified in *S. cerevisiae*, mammals, and other organisms [14], is a pathway that signals decreased mitochondrial functionality to the nucleus, where it activates expression of specific genes. Activation, by RTG gene-dependent retrograde signaling (RTG signaling), of expression of genes (such as *CIT2*) involved in anaplerotic pathways was described many years ago in yeast cells grown in liquid cultures [15, 16]. However, we recently showed that RTG signaling in colonies is more complex than that described previously and activates expression of different genes in differentiated cells [17]. We identified three branches of RTG signaling that are specific to U cells (the Ato branch), the upper subpopulation of L cells (the Cit2 branch), and the lower subpopulation of L cells (the cell-viability branch). These signaling branches regulate different gene targets and/or contribute differently to viability of each of the three subpopulations [17].

To extend our knowledge of the similarities and differences of differentiated colony cells and, in particular, the dynamics of their formation, we performed detailed genome-wide transcription profiling by RNA sequencing of cell subpopulations isolated from different areas of acidic- and alkali-phase colonies. We show that upper cells are unique in terms of transcription changes in time and space and also that U cells are a rather homogeneous subpopulation from the point of the view of transcription. Although U cells have significant expression similarities with cells localized to marginal regions, several differences, including those related to mitochondrial functions, contribute to their unique properties. In contrast, lower cells exhibit little temporal transcription dynamics. Furthermore, L cells appear to consist of two subpopulations that differ dramatically in expression, the upper one being in some ways similar to U cells. Altogether, these new findings point towards additional, yet to be identified levels of colony complexity and support the hypothesis of an important role for mitochondria and related signaling in the differentiation of ageing colonies and the escape of specific subpopulations from the stress caused by nutrient depletion.

## 2. Results and Discussion

### 2.1. Genome-Wide Transcription Profiling of Cell Subpopulations Separated from 6- and 15-Day-Old Colonies

**Figure 1:**
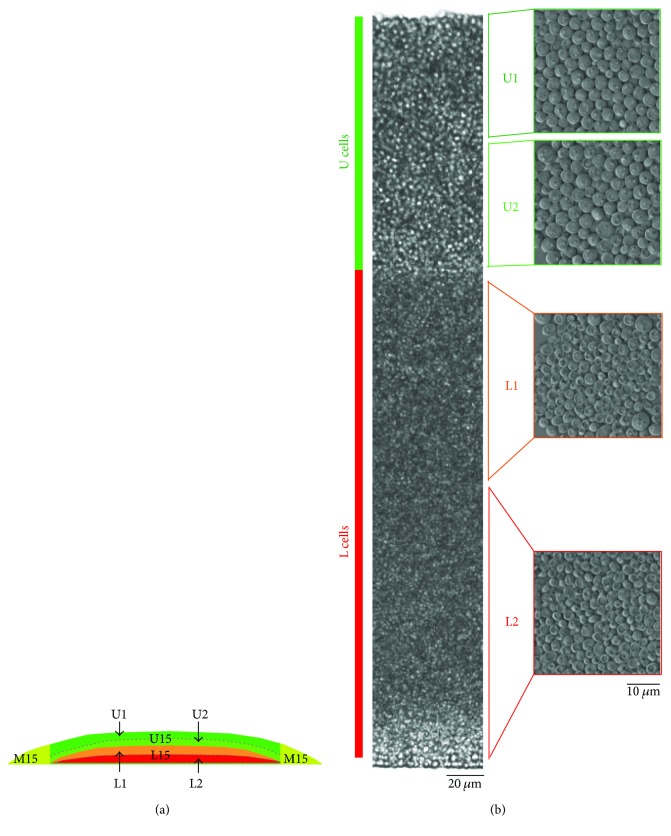
Subpopulations in 15-day-old colonies. (a) Schematic of subpopulations in 15-day-old colonies. (b) Small subpopulations separated from the central part of the colony. (left) Vertical cross section of a 15-day-old colony. (right) Cell subpopulations separated from the colony for RNA sequencing. Cells were visualized by Nomarski contrast. U: upper; L: lower; U1: upper U cells; U2: lower U cells; L1: upper L cells; L2: lower L cells.

Three cell subpopulations were separated from 15-day-old alkali-phase giant colonies grown on GMA agar: U cells from the central upper (U15 cells) parts of the colonies, L cells from the central lower (L15 cells) parts of the colonies, and cells that localize to marginal regions (M15 cells) (Figure 1(a)). Similarly, we also separated 6-day-old acidic-phase colonies, that is, colonies in the stage before U/L cell differentiation, into 3 cell subpopulations: cells from the upper (U6 cells) and cells from the lower (L6 cells) regions of the colony centre and cells from the colony margin (M6 cells). To study the composition of fully differentiated U15 and L15 cells, we further separated both U15 and L15 cells into two smaller cell subpopulations: U1 cells from upper and U2 cells from lower layers of U15 cells and L1 cells from upper and L2 cells from lower layers of L15 cells (Figure 1(b)). Total RNA extracted from these ten cell subpopulations was used for RNA sequencing. Altogether, we sequenced 30 transcriptomic libraries, representing three biological replicates of these ten subpopulations (see Materials and Methods for details).

**Figure 2:**
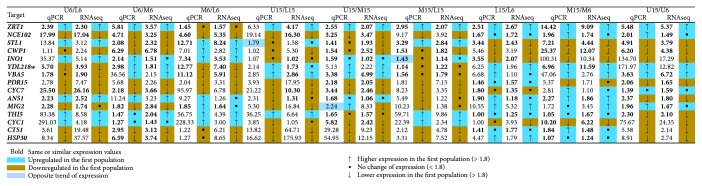
RT-qPCR verification of RNA-seq results. RNA-seq results for selected genes were verified using RT-qPCR. First-strand cDNA was synthesized from total RNA using random primers and RT-qPCR carried out in triplicate on 5-fold diluted cDNA. ΔΔ*C*_T_ values were normalized to those of housekeeping genes (up to 5 genes) to compare fold differences between samples.

Differential expression (DE) was detected for 7055 loci, reflecting expression differences among the differently localized cell subpopulations and changes in major subpopulations during colony development. Small fold transcription differences between cell types (even if statistically significant in robust sequencing data) usually do not reflect a significant difference in protein/metabolite levels, so we restricted our analysis of differential expression to those genes with an adjusted *p* value below 0.05 (*p*_adj_ < 0.05) with expression fold changes of 1.8 or greater. Across 31 comparisons, we found 43,488 differential expressions (DE) involving 5036 unique protein-coding genes fulfilling these criteria. Of these DEs, 32,898 (76%) retain significance when applying the most stringent Bonferroni correction (*p* value < 4.8*E*−06, [18]). The equivalent results for lncRNA are 11,354 (*p*_adj_ value) and 3599 (Bonferroni correction). The differences in expression of selected genes were confirmed by RT-PCR (Figure 2).

### 2.2. Differential Expression in Upper, Lower, and Margin Subpopulations and Functional Analysis

**Figure 3:**
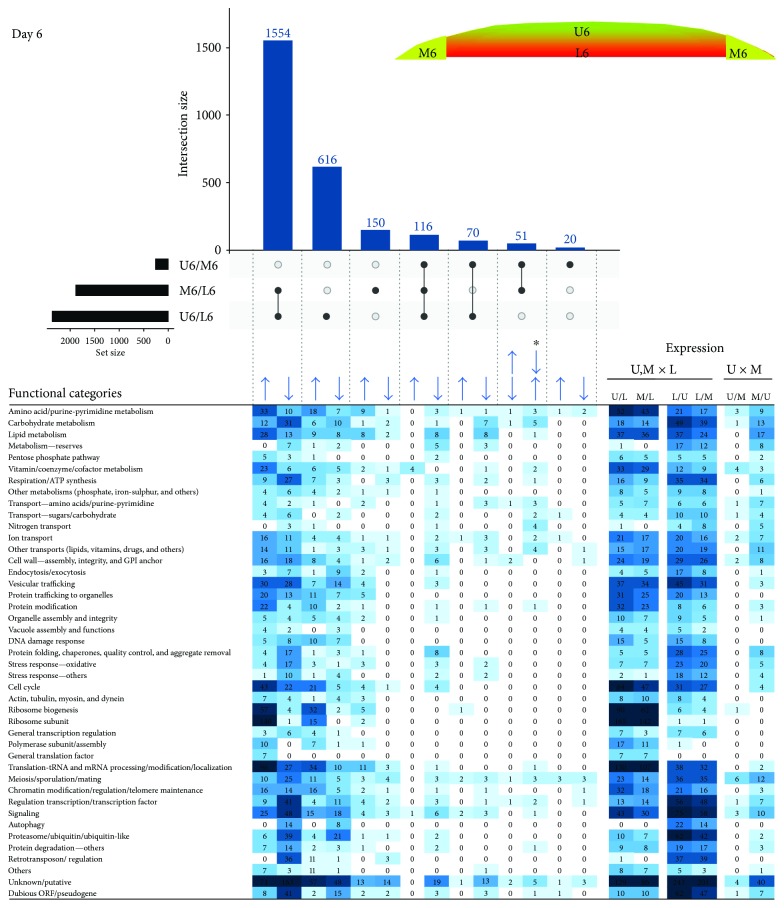
Comparing cell subpopulations in 6-day-old colonies. UpSet plot of datasets of DE genes in upper (U6), lower (L6), and marginal (M6) cells from 6-day-old colonies (upper part). Horizontal black bar chart indicates numbers of genes, DE in each individual comparison. Intersect “connectors” indicate comparisons in which a given number of genes (vertical blue bar chart) were DE. Only major intersections are shown. Heat map of genes assigned to functional categories and clustered according to FC and DE in different sample comparisons (lower part). Number in heat map cell = number of genes from FC, up- or downregulated in sample comparison. The higher the number of up-/downregulated genes, the more intense the color. Arrows indicate upregulation/downregulation in the respective subpopulation ratio(s); asterisk indicates categories of genes that are differently up-/downregulated in the respective subpopulation ratios.

**Figure 4 fig4:**
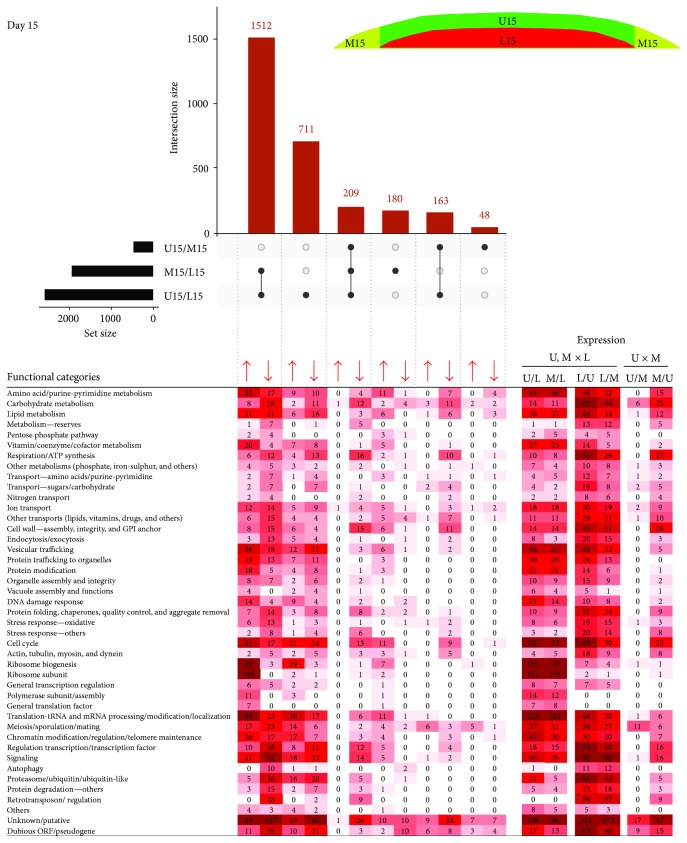
Comparing cell subpopulations in 15-day-old colonies. UpSet plot of datasets of DE genes in upper (U15), lower (L15), and marginal (M15) cells from 15-day-old colonies (upper part). Only major intersections are shown. Heat map of genes assigned to functional categories and clustered according to FC and DE in different sample comparisons (lower part). Number in heat map cell = number of genes from FC, up- or downregulated in sample comparison. The higher the number of up-/downregulated genes, the more intense the color. Arrows indicate upregulation/downregulation in the respective subpopulation ratio(s).

**Figure 5:**
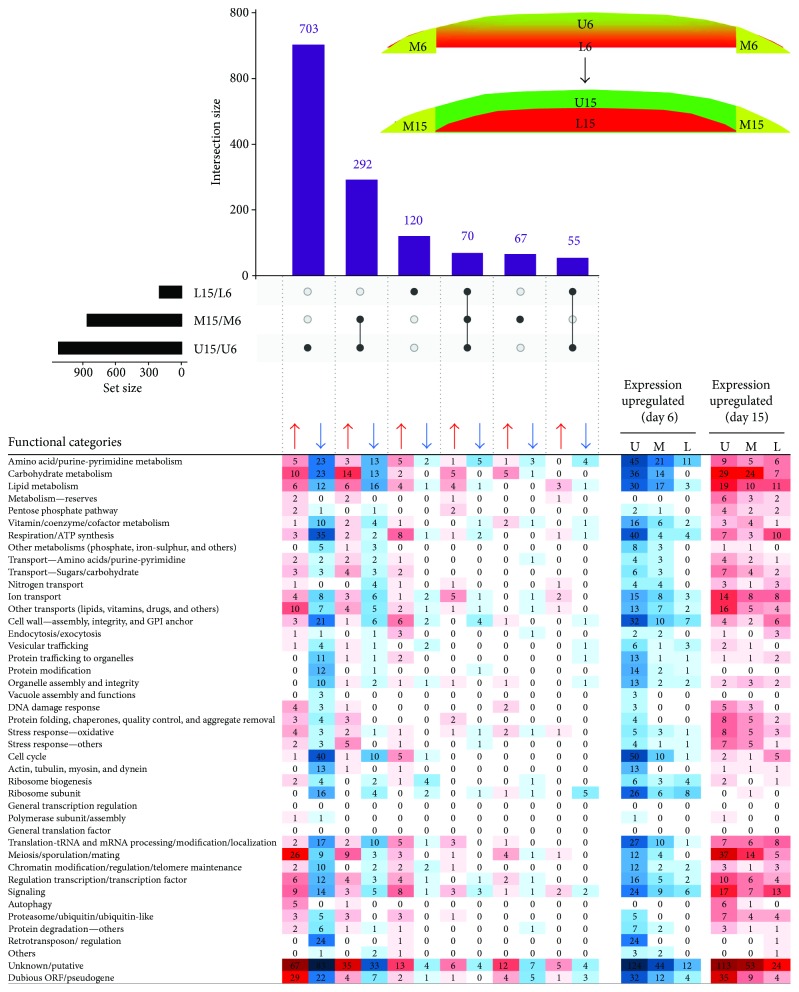
Time point comparisons. UpSet plot of datasets of DE genes in upper (U6 and U15), lower (L6 and L15), and marginal (M6 and M15) cells from 6- and 15-day-old colonies, respectively (upper part). Only major intersections are shown. Heat map of genes assigned to functional categories and clustered according to FC and DE in different sample comparisons (lower part). The higher the number of up-/downregulated genes, the more intense the color. Number in heat map cell = number of genes from FC, up- or downregulated in sample comparison. Red cell highlighting: FC upregulated at day 15 relative to day 6. Blue cell highlighting: FC upregulated at day 6 relative to day 15. Arrows indicate upregulation/downregulation in the respective subpopulation ratio(s).

Differentially expressed genes were identified in pairs of compared cell subpopulations. To assess overall differences/similarities between the subpopulations, we compared datasets of DE genes using Intervene's UpSet module [19], which visualizes the intersection of multiple datasets in UpSet plots. For clarity, we compared subpopulations of 6- and 15-day-old colonies separately, and, to estimate time-differences, we also compared cells localized to the same position in 6- and 15-day-old colonies (Figures 3 [Fig fig4]–5). Comparison of both U6, L6, and M6 cells (Figure 3) and U15, L15, and M15 cells (Figure 4) revealed at both developmental times the most prominent expression differences between upper and lower cells (2346 and 2594 genes, resp.) and between margin and lower cells (1861 and 1900 genes, resp.). Differences between upper and margin cells were moderate and more prominent in 15-day-old (420 genes) than in 6-day-old (257 genes) colonies, indicating that upper and margin subpopulations are similar to each other but different from lower cells. Upregulated genes common to both upper and marginal cells relative to lower cells numbered 1554 (6 days) and 1512 (15 days) genes. Unique differences in expression between upper and lower cells were represented by 616 DE genes at day 6 and 711 genes at day 15. Those genes uniquely differing between margin and lower cells numbered 150 genes at day 6 and 180 genes at day 15. Only a few unique DE genes were identified between upper and margin cells in both times (20 and 48 genes, resp.).

Examination of temporal changes in respective colony regions showed (Figure 5) that major changes occur during differentiation from upper cells of 6-day-old colonies to U cells of 15-day-old colonies (1120 DE genes, 703 of which were unique to upper cells). In marginal cells, temporal changes concern the expression of 429 genes, 292 of which were changed in both upper and marginal cells and 67 unique to marginal cells. Temporal changes in lower cells were relatively moderate and regard 245 DE genes, 120 of which were unique to lower cells and 55 jointly changed in lower and upper cells.

**Figure 6:**
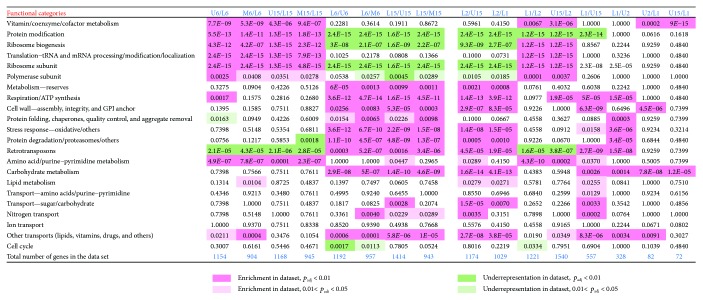
Statistical analysis of FC enrichment/underrepresentation among DE genes. Fisher's exact test was used to determine whether the percentage of genes in an upregulated dataset that was mapped to a particular FC was significantly higher or lower than the percentage of genes in the yeast genome that was mapped to the same functional category. *p* values, adjusted for multiple testing using the Benjamini-Hochberg procedure (*p*_adj_), are shown for selected FCs of genes, up- or downregulated in individual sample comparisons. Significantly enriched: pink (FDR 0.05) and dark pink (FDR 0.01). Significantly underrepresented: green (FDR 0.05) and dark green (FDR 0.01).

Subsequently, we performed global functional grouping of DE genes using gene ontology analysis and hierarchical clustering, further controlled by manual assessment of individual gene functions based upon information in the SGD (http://www.yeastgenome.org/) and the literature. This analysis clustered genes to different functional gene categories (FC) that were upregulated in upper, lower, and margin cell subpopulations as well as during temporal changes in cells localized to particular positions (Figures 3 [Fig fig4]–5). It is worth noting here that the term “upregulation”/“downregulation” of gene expression used throughout the text is usually relative to the opposite subpopulation and does not imply that a gene expression difference is due to an increased rate of transcription in one subpopulation or decreased rate in the other. This functional categorization of DE genes helped us to further identify similarities/differences among the subpopulations and their developmental changes. In parallel with this cluster analysis, we performed statistically supported enrichment analysis of functional categories in our datasets, which confirmed FC enrichment (compared with the genome) in most cases (Figure 6).

In both 6- and 15-day-old-colonies, several prominent FCs were similarly up- and downregulated in upper and margin cells versus lower cells. This finding indicates that cell diversification leading to fully differentiated U and L cells in 15-day-old colonies has already begun in younger colonies, in which cells cannot be clearly distinguished according to morphology. Prominent FCs, the genes of which were upregulated in both upper and margin cells when compared with lower cells at both time points, include genes with functions in vitamin/cofactor metabolism, protein modifications, ribosome biogenesis, translation and tRNA/mRNA processing and modifications, or encoding ribosome or polymerase subunits (Figures 3 and 4). On the other hand, lower cells at both time points upregulated genes involved in metabolism of reserves (storage compounds), respiration and mitochondrial ATP synthesis, protein folding and protein quality control, oxidative and other stress responses, proteasome functions, and other protein degradation genes and retrotransposons. The repression of polymerase, ribosome and protein biosynthesis genes on one hand, and induction of protein folding and degradation, respiration and stress response genes on the other, in lower cells compared with upper or marginal cells, is typical of cells subject to environmental stress or the diauxic shift [20, 21]. These data are in agreement with the higher level of reactive oxygen species and other stress-related features of L cells [6] and may indicate that lower cells are already more stressed than upper cells in 6-day-old colonies. Other large FCs of genes, DE at both day 6 and day 15, include genes involved in amino acid, carbohydrate, and lipid metabolism; genes for different transporters; and genes involved in the cell cycle. Each of these FCs is not typical of any particular subpopulation; it includes some genes upregulated in upper/margin cells but also other genes upregulated in lower cells. Often, these FCs also include genes differentially expressed between days 6 and 15 (Figure 5), indicating high spatiotemporal dynamics in expression of genes involved in these cellular processes. Enrichment analysis (Figure 6) confirmed most of the FCs that were differently expressed between different cell types: FCs that are enriched against the background in U/M versus L cell comparisons include those involved in vitamin/cofactor metabolism, protein modification, ribosome biogenesis/subunits, translation, polymerase subunits, and amino acid/purine/pyrimidine metabolism. FC groups that are enriched in L versus U/M cell comparisons include those involved in cell wall, respiration/ATP synthesis, protein folding, stress response, protein degradation, carbohydrate metabolism, other transports and retrotransposons. Interestingly, some FCs (mainly protein modification and ribosome biogenesis/subunit FCs) are significantly underrepresented underrepresented among genes upregulated in L cells versus U and M cells not only that genes from the functional category are repressed but that the functional category itself is generally repressed in L cells versus U or M cells.

### 2.3. Expression Differences among Small Subpopulations of U and L Cells from Differentiated 15-Day-Old Colonies

**Figure 7:**
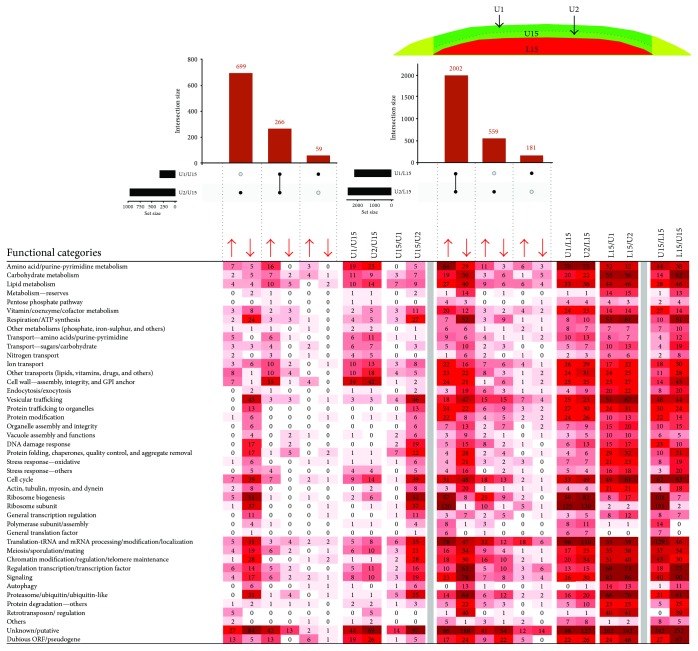
Comparing small upper-cell subpopulations. UpSet plot of datasets of DE genes in small upper-cell subpopulations (U1 and U2) and upper (U15) and lower (L15) cells from 15-day-old colonies (upper part). Only major intersections are shown. Heat map of genes assigned to functional categories and clustered according to FC and DE in different sample comparisons (lower part). Number in heat map cell = number of genes from FC, up- or downregulated in sample comparison. The higher the number of up-/downregulated genes, the more intense the color. Arrows indicate upregulation/downregulation in the respective subpopulation ratio(s).

In addition to comparing major subpopulations, differential expression results were collected from comparisons of expression data of U1, U2, L1, and L2 subpopulations separated from 15-day-old colonies (Figure 1). At first glance, these comparisons revealed relatively large expression differences between L1 and L2 cells, whereas no DE genes fulfilling our criteria (*p*_adj_ < 0.05; fold change 1.8) were identified between U1 and U2 subpopulations, indicating that in contrast to L cells, U cells are relatively homogeneous. For this reason, we extended our analyses and compared all small subpopulations with U15 and L15 datasets comprising results for major colony subpopulations. Dataset comparisons were then analyzed using Intervene's UpSet module and gene ontology (GO) functional clustering as indicated above. Further statistical analysis confirmed the enrichment of FCs in selected datasets versus the genome (Figure 6). The data obtained provided us with a complex view of the differences/similarities among the subpopulations, as summarized in Figures 6 and 7 and described below.

Comparison of U1 and U2 cells with L15 cells (Figure 7) revealed that most of the genes differentially expressed between U1 and L15 and U2 and L15 are the same 2002 genes. 1627 of these genes were also differentially expressed between U15 and L15 cells. Functional categorization of DE datasets U1/L15 and U2/L15 and their comparison with the U15/L15 dataset showed a high level of FC similarity among both up- and downregulated genes. As expected, comparison of U1 and U2 cells with U15 cells revealed a much lower total number of DE genes (1290 genes) than comparison of U1 and U2 cells with L15 cells (4744 genes) and showed higher expression differences between U2 and U15 (965 genes) than between U1 and U15 (325 genes) cells. Functional categorization of the datasets showed 242 genes upregulated and 83 genes downregulated in U1 versus U15 cells. Of the upregulated genes, 204 genes overlapped with genes upregulated in U2 versus U15 cells. In addition to the largest category of unknown genes/dubious ORFs (55 genes), genes of amino acid, carbohydrate and lipid metabolism (altogether 40 genes), transporter genes (36 genes), and genes related to cell wall function (35 genes) fall into this group. Differences between U2 and U15 were more prominent and regard 336 upregulated and 629 downregulated genes. The largest functional category of unique U2-upregulated genes (132 genes) again includes unknown/dubious genes (40 genes). Interestingly, some groups of genes, repressed in U2 versus U15 (567 genes), comprise FCs also typically repressed in L15 versus U15, such as genes for ribosomal subunits (37 genes), ribosome biogenesis (51 genes), translation, and tRNA/mRNA modification (31 genes). On the other hand, other genes repressed in U2 cells belong to functional categories repressed in U15 versus L15, such as genes involved in respiration (24 genes) and proteasome function (31 genes). These data indicate that U2 cells have more marked downregulation of some functions typically repressed in all U cells, such as respiration. In addition, U2 cells activate, to a lesser extent than U1 cells, some FCs typically upregulated in U15 versus L15 cells, such as those involved in ribosome functions and translation. These data also confirmed that expression differences observed between U2 and L15 cells are not simply caused by U2 sample contamination by L cells.

**Figure 8:**
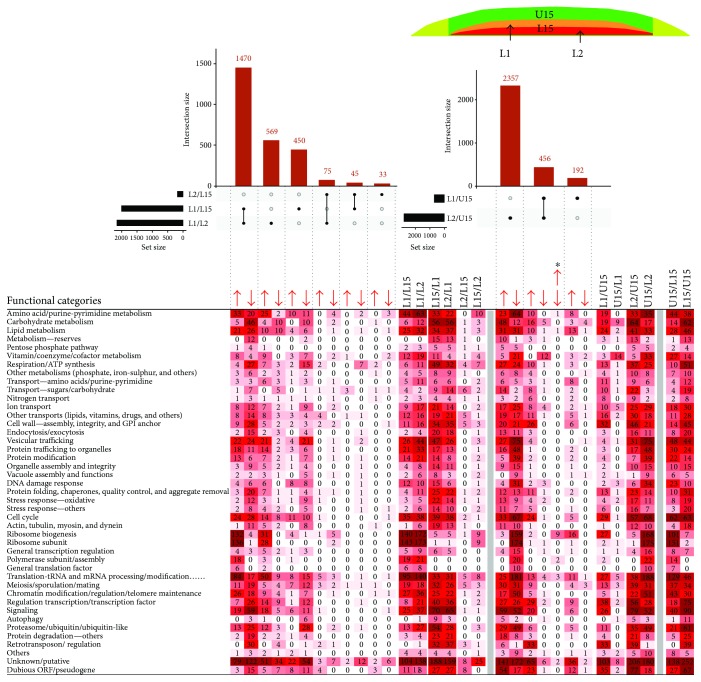
Comparing small lower-cell subpopulations. UpSet plot of datasets of DE genes in small lower-cell subpopulations (L1 and L2) and upper (U15) and lower (L15) cells from 15-day-old colonies (upper part). Only major intersections are shown. Heat map of genes assigned to functional categories and clustered according to FC and DE in different sample comparisons (lower part). Number in heat map cell = number of genes from FC, up- or downregulated in sample comparison. The higher the number of up-/downregulated genes, the more intense the color. Arrows indicate upregulation/downregulation in the respective subpopulation ratio(s); asterisk indicates the category of genes that are differently up-/downregulated in the respective subpopulation ratios.

**Figure 9:**
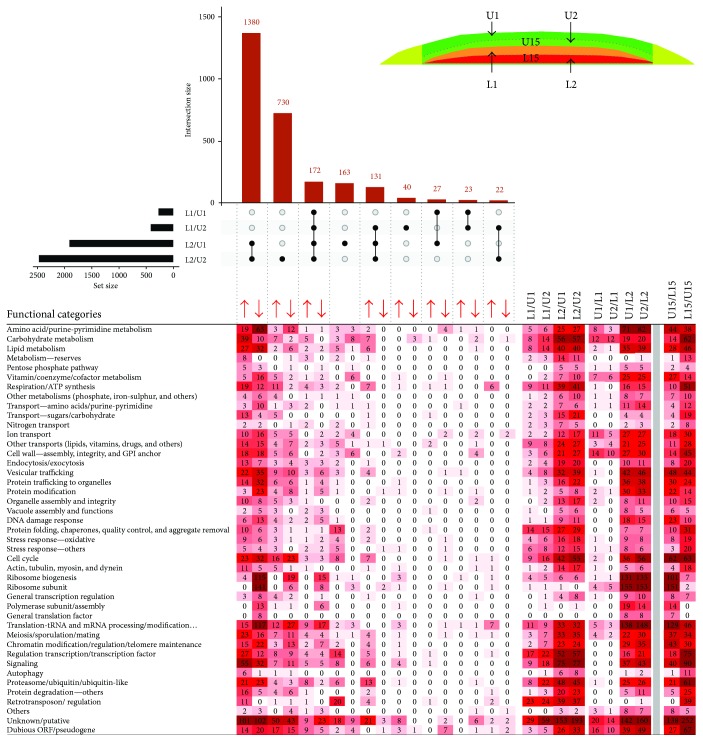
Comparing small upper- and lower-cell subpopulations. UpSet plot of datasets of DE genes in small lower-cell (L1 and L2) and small upper-cell (U1 and U2) subpopulations from 15-day-old colonies (upper part). Only major intersections are shown. Heat map of genes assigned to functional categories and clustered according to FC and DE in different sample comparisons (lower part). Number in heat map cell = number of genes from FC, up- or downregulated in sample comparison. The higher the number of up-/downregulated genes, the more intense the color. Arrows indicate upregulation/downregulation in respective subpopulation ratio(s).

Comparison of L1 and L2 expression data identified 2144 DE genes, indicating prominent differences in these two cell subpopulations (Figure 8). Further comparison of L1 and L2 with L15 and U15 expression datasets revealed that expression characteristics of L2 cells are similar to those of L15. L2 and L15 cells differ in expression of only 184 genes, whereas, as expected, comparison of L2 and U15 datasets identified 2813 DE genes, which is a number similar to that for the L15/U15 comparison (2595 DE genes). On the other hand, comparison of L1 and L15 datasets identified 1997 DE genes, whereas comparison of L1 and U15 datasets identified only 648 DE genes, indicating more similarities between L1 and U15 than between L1 and L15. Mutual comparison of expression datasets of L1 and L2 with U1 and U2 further supported this conclusion (Figure 9). To gather more information about metabolic differences between L1 and L2 cell subpopulations, we performed functional categorization of expression datasets and compared them with the L15 and U15 functional groups (Figure 8). Comparison of FCs of genes differentially expressed among L1, L2, U15, and L15 confirmed similarity between L2 and L15 functional datasets. Differences between L2 and L15 datasets were small and, apart from unknown genes (41 genes), regard only individual genes spread among different functional groups. In accordance, comparison of L2 with U15 showed a similar profile of up- and downregulated FCs to the comparison of L15 and U15 (Figure 8). Functional categorization of the large number of genes differentially expressed in L1 versus L15 showed an extensive overlap with FCs differentially expressed in L1 versus L2 (1470 genes, 742 up- and 728 downregulated) as well as overlap with FCs up- and downregulated in U15 versus L15 cells (1616 genes, 654 up- and 962 downregulated) (Figure 8) supporting the prediction that L1 cells, in some metabolic aspects, resemble U15 cells. Comparison of L1 and U15 datasets further revealed 72 downregulated genes and 575 upregulated genes; 366 of the latter being also upregulated in L2 versus U15. These data show that cells in upper layers of the L cell subpopulation repress almost no specific genes when compared with U cells, but they increase expression of many L cell typical genes. L cell genes that are upregulated in both L1 and L2 versus U15 cells include genes involved in amino acid, carbohydrate and lipid metabolism, and respiration (altogether 46 genes); genes for several transporters (33 genes); and genes involved in cell wall function (26 genes), protein folding (11 genes), cell cycle (24 genes), signaling and transcription regulation (49 genes), and retrotransposons (33 genes). However, in addition, L1 cells versus U15 partially upregulate FCs comprised of genes that are typically downregulated in L15 cells versus U15 cells, such as genes involved in ribosome biogenesis (27 genes, 24 of which are downregulated in L2 versus U15 and/or L1) and translation and tRNA/mRNA modification (27 genes, 13 of which are downregulated in L2 versus U15 and/or L1). 72 genes downregulated in L1 versus U15 are dispersed across many FCs, apart from a cluster of 14 genes involved in vitamin/cofactor metabolism. As expected, FCs upregulated in L1 versus U1 and L1 versus U2 (Figure 9) are similar to those upregulated in L1 versus U15. Interestingly, some FCs such as amino acid and lipid metabolism, cell cycle, signaling, proteasomal function, and unknown gene groups include more upregulated genes in L1 versus U2 than in the L1 versus U1 datasets. In accordance with this, the total number of upregulated genes is higher between L1 and U2 (329 genes) than between the L1 and U1 (208 genes) datasets. This indicates slightly greater expression differences between U2 and L1, despite these subpopulations being localized more closely within the colony than L1 and U1.

### 2.4. Spatiotemporal Gene Expression in Ageing Colonies

**Figure 10:**
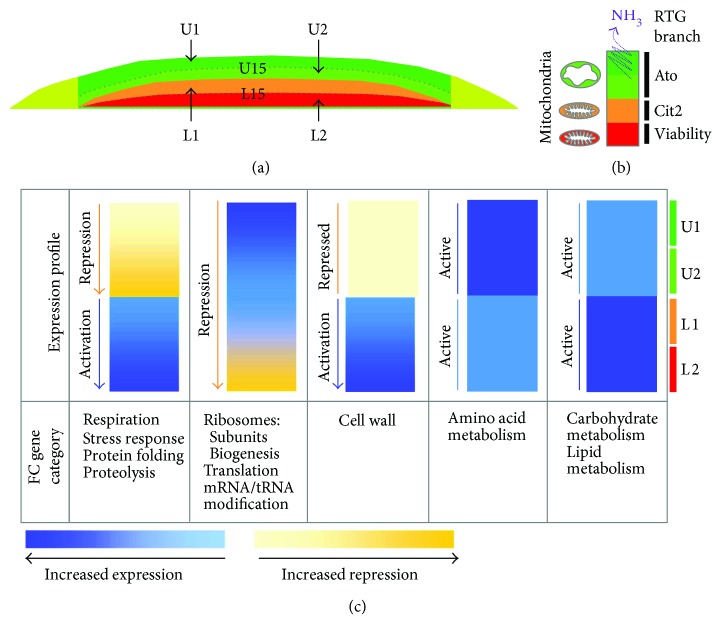
Functional category (FC) expression through a yeast colony. (a) Diagram of 15-day-old colony subpopulations analyzed by RNA-seq in this study. (b) Overview of RTG regulatory pathway branches that are functional in fully differentiated 15-day-old colonies [17, 39]. (c) Based on the differential expression of constitutive genes in different population/subpopulation comparisons in 15-day-old colonies, a schematic was produced indicating expression/repression of various FCs through the colony cross-section from uppermost (U1) to lowest (L2) subpopulation in the colony centre. Increased color density corresponds to increased activation (blue) or repression (yellow) as indicated in bars below the pictures.

The expression data obtained provide an in-depth view of the functional gene groups, differentially expressed in time and space. In the following paragraphs and in the model (Figure 10), we summarize the major conclusions from these comparisons.

Comparison of the three major cell types (upper, lower, and margin cells) in young acidic-phase and older fully differentiated alkali-phase colonies clearly revealed the most prominent temporal changes in cells localized to upper colony layers (1120 genes), that is, in cells that differentiate during the colony transition from acidic- to alkali-phase into U cells, and which exhibit specific physiology and regulation as described previously [6, 13, 17, 22]. Most of these temporal changes regard genes whose expression changes neither in margin cells nor in lower cells during ageing (~700 genes). Interestingly, about twice as many of these U cell unique genes were repressed than activated in 15-day-old colonies compared to upper cells of younger, 6-day-old acidic-phase colonies. The repressed genes include those involved in respiration, that is, in a process previously shown to be dampened in U cells [6, 13], and are likely involved in determining some of the specific properties of U cells, such as activation of individual branches of RTG signaling subsequently causing changes in gene expression [17]. In contrast to upper cells, many fewer temporal expression changes were observed at the colony margin (442 genes) despite the fact that these cells exhibit extensive similarities with U cells as shown by upper-/margin-/lower-cell comparisons in both developmental times. In addition, most of the expression changes occurring in marginal cells between days 6 and 15 also occur in upper cells or in both upper and lower cells over the same time period. Unique temporal changes in margin cells thus concern only ~70 genes, that is, ten times fewer genes than changes in U cells. The lowest number of temporal expression changes occur in lower cells (258 genes in total), but, as in upper cells, many of these changes (~120 genes) involve unique genes that do not change expression in upper and marginal cells during the time period. In contrast to U cells, three times as many of these unique DE genes were upregulated than downregulated in L cells of 15-day-old colonies. These findings indicate that in contrast to U cells (and, to a lesser extent, also to L cells), marginal cells, which are the most efficiently growing cell subpopulation even in older colonies [23], undergo almost no margin-cell-type-specific changes during colony ageing.

Temporal differences in gene expression of upper, lower, and marginal cells were in agreement with observed differences among these cell subpopulations at individual time points. At both time points (day 6 and day 15), the most prominent cell-type-specific differences were seen between upper and lower cells, where 616 differentially expressed genes were identified only in U6 versus L6 and 711 only in U15 versus L15 cells. On the other hand, a much smaller number of genes exhibited differential expression uniquely between marginal and lower cells (150 and 180 genes at day 6 and day 15, resp.) and negligible expression differences were identified uniquely between upper and marginal cells (20 and 48 genes at days 6 and 15, resp.). The most prominent differences occurring among subpopulations at both time points concerned genes differentially expressed between lower cells and the rest of the colony (i.e., both upper and marginal cells) (1554 and 1512 genes at days 6 and 15, resp.). This finding argues that the greatest similarity exists between upper and marginal cells, which is in accordance with the observation that several protein markers of U cells are also produced in marginal cells, but not in L cells [6, 13, 24]. Although 2.5 times fewer genes were DE relative only to U cells as relative to both U and M, several important FC categories were overrepresented in the former dataset. Examples include genes involved in cell wall function, proteasomes, and meiosis and among U cell-repressed genes involved in respiration, vitamin metabolism, vesicular transport, and cell cycle.

Comparison of small subpopulations derived from U cells (U1 and U2 cell) and from L cells (L1 and L2), to each other and with U15 and L15 cells, provided additional insight into colony complexity (Figure 10). L1 and L2 cells, although morphologically similar, with large vacuoles and other typical characteristics [6, 13, 17], exhibited distinct profiles of DE genes indicating that these cell subpopulations differ significantly. Whereas L2 cells are similar to L15 cells, L1 cells differ from both L2 and L15 in many parameters similar to differences between U15 and L15; L1 cells were thus, to some extent, similar to U15 cells. The L1 to U15 similarity was particularly evident when comparing L1 versus U15 repressed genes, among which only one prominent functional category of genes (involved in vitamin metabolism) was detected. On the other hand, genes belonging to several FCs typically upregulated in L15 cells were also upregulated in L1 cells, including the categories of respiration, cell wall function, protein folding, proteasomes, and retrotransposons. Thus, L1 cells combine several L cell-specific features with some features typical of U cells, such as expression of many genes encoding ribosome subunits or involved in ribosome biogenesis, translation, and tRNA/mRNA modification. Similar to U and L cells, functional categories of metabolic genes (amino acid, carbohydrate, and lipid metabolism) are active in both L1 and L2 cells and differ regarding the expression of individual genes. In contrast to L1 and L2 cells, differences between U1 and U2 cells were small and no DE genes were identified by direct comparison of these subpopulations. However, comparison of U1 and U2 subpopulations with larger subpopulations of U15 and L15 cells as well as with L1 and L2 revealed some significant differences. The identified U1 and U2 differences in particular concern FCs of genes that are repressed in U2 versus U15 (but not repressed in U1 versus U15). These FCs include on one hand those that are also typically repressed in U15 cells versus L15 cells, such as respiration, protein folding, and proteasomal functions, but on the other hand, also those that are typically repressed in L15 cells versus U15 cells, such as ribosomal subunits or ribosome biogenesis, translation, and mRNA/tRNA modification. Hence, these data show that U2 cells repress some genes similarly to L cells but that, on the other hand, some processes typically repressed in U cells but active in L cells, such as respiration, are more effectively repressed in U2 cells than in U1 (and U15) cells.

### 2.5. Respiration and Stress-Related Process Exhibit Similar Spatiotemporal Profiles within Developing Colonies

**Figure 11:**
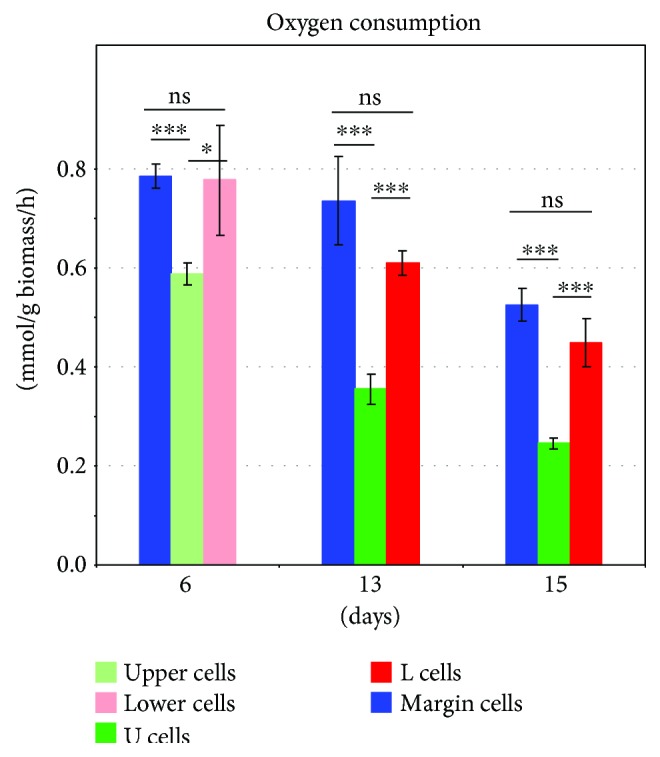
Oxygen consumption by cell subpopulations from 6-, 13-, and 15-day-old colonies. The mean of four biological replicates is shown ±SD. *t*-test *p* values of 0.05 or less were considered statistically significant: ^∗^ *p* < 0.05 and ^∗∗∗^ *p* < 0.001; ns: not significant.

Previous study of the dynamics and development of fully differentiated and morphologically distinct U and L cells [6] showed that the differentiation process lasts several days, starting at about 9-10 days and resulting in fully developed and sharply separated U and L cell layers at ~day 14. Here, we show that metabolic diversification, especially of cells located in upper colony parts, has already started in younger colonies that are in late acidic phase and formed of cells that are still morphologically relatively homogeneous. This concerns particularly the expression of genes involved in ribosome function and translation in upper cells, and genes for proteasomal functions, retrotransposons, and stress response in lower cells of 6-day-old colonies. These differences persist and become more pronounced in fully differentiated U and L cells of 15-day-old colonies, despite the large variation in expression of individual genes being observed within these groups over time. This finding shows that cellular features are to a certain extent determined by cell position within the colony as early as late acidic phase, in which colonies are still linearly growing [23]. This finding also shows that in colonies as young as 6 days, cells located in lower positions closer to agar with better access to nutrients than upper cells already exhibit some features of starvation and stress, such as activation of heat shock genes, stress-defense genes, and genes for proteasomes. Concurrently, colony cells start to diversify the expression of FCs including genes involved in oxidative phosphorylation (OXPHOS) and in respiration-dependent ATP synthesis in mitochondria. When compared with lower cells, most of the DE genes belonging to this FC are downregulated in both upper and marginal cells, indicating reduced mitochondrial activity, despite the facts that both upper and marginal cells have good access to air (and oxygen), that the colonies were grown on complete respiratory medium, and that marginal (and to some extent upper) cells are still dividing in colonies during this developmental stage. Thus, already in 6-day-old colonies, mitochondria may be less active in upper cells that do not exhibit stress features, than in lower cells that are becoming stressed. Simple oxygen consumption experiments (Figure 11) confirmed decreased oxygen consumption by upper cells relative to lower cells of 6-day-old acidic-phase colonies, but did not identify differences between margin and lower cells.

Later on in colony development, U cells of 15-day-old alkali-phase colonies downregulate most of the DE genes of the OXPHOS/ATP synthesis functional category, compared with L cells. This is in agreement with previous findings concerning the differences in mitochondrial morphology and oxygen consumption measured in separated U and L cells as well as in OXPHOS gene expression determined by microarrays [6, 13]. However, the current study revealed a more complex view of the expression of OXPHOS/ATP synthesis genes in differentiated U, M, and L cells and their subpopulations. Expression of these genes was observed in the following degrees: U15 < M15 < L15. Oxygen consumption experiments (Figure 11) confirmed reduced oxygen consumption by U cells compared with both L and M cells of 15-day-old colonies but, similarly to 6-day-old colonies, did not identify significant differences between M and L cells. Time-line comparison of cells from 6-, 13-, and 15-day-old colonies showed, in addition, a gradual decrease in oxygen consumption by all subpopulations as colonies aged. Transcriptomic comparison of smaller subpopulations showed that U2 cells (which are localized nearer to L1 cells) are the subset of U cells that exhibits the absolutely lowest expression level of OXPHOS/ATP synthesis genes, whereas L2 cells exhibited highest expression (Figure 10(c)). Whether these differences are reflected in differences in oxygen consumption remains to be resolved as our currently used measurements are not suitable for tiny subpopulations. The observed pattern of expression of the FC including OXPHOS/ATP synthesis genes overlaps with that of other FC groups, including a group of stress-related genes (genes involved in protein folding and stress response). This in turn indicates that metabolic reprogramming, leading to a decrease in respiration when nutrients start to be limited (as what happens during colony ageing), may alleviate the stress. Our new data also indicate that the cells at the boundaries of U and L cells (i.e., U2 and L1) differ significantly in the expression of genes belonging to these FCs, as they differ in other prominent features, including cell morphology which shows a sharp demarcation border between U and L cells [6, 13, 17]. On the other hand, the data indicate that at least some of the FCs that are already upregulated in upper cells in late acidic-phase colonies and that continue to be upregulated in alkali-phase colonies, such as FCs related to ribosome functions and translation, exhibit a gradual gradient-like pattern of expression, being most highly upregulated in the upper-most colony cell layers (U1 cells) and decreasing expression towards lower-cell layers (L2 cells) (Figure 10(c)).

In summary, the major differences between cells located near the demarcation border between morphologically distinct U and L cells represent functional gene categories that are upregulated in all L cells and repressed most in U2 cells, such as genes involved in respiration. Expression data of these FCs indicate a gradient of gene repression in U cells in the direction U1 → U2 and conversely a gradient of gene activation in L cells in the direction L1 → L2. On the other hand, L1 cells do not differ dramatically from U2 cells in terms of the expression of functional groups typically upregulated in U cells, such as groups of ribosome and translation-specific genes. The observed pattern of expression of OXPHOS/ATP synthesis genes in 15-day-old colonies indicates that mitochondrial properties may differ not only among U and L cells as described previously [6, 13], but potentially also among the cell layers within these subpopulations. Subsequently, divergent mitochondria may be involved in different cellular processes including the activation of different branches of RTG signaling that contribute to the colony differentiation processes [17]. A link between mitochondrial properties and RTG pathway specificities is also supported by the observation that, despite OXPHOS/ATP synthesis gene expression and oxygen consumption being lowest in upper cells (compared with both lower and marginal cells), oxygen consumption and the expression of genes of this FC differ between days 6 and 15: OXPHOS/ATP synthesis genes are more highly repressed and oxygen consumption reduced in U15 cells (that activate the Ato branch and in parallel repress the Cit2 branch of RTG signaling) compared to U6 cells that have an active Cit2 branch. In addition, the expression of respiratory genes is highest in L2 cells in 15-day-old colonies, that is, in a subpopulation that activates the cell-viability branch of RTG signaling (unrelated to Cit2 and Ato branches) that is necessary for longevity of L2 cells (Figure 10(b) and [17]). Nevertheless, expression data and physiology data do not always correlate, as demonstrated by margin and lower-cell comparisons. Further analyses of the biochemistry and activities of mitochondria from differentiated cell subpopulation, and of their relationship with other specific cellular properties, are therefore needed to demonstrate potential functions of differently altered mitochondria in RTG signaling and in other cellular processes during the colony differentiation process.

## 3. Conclusions

Genome-wide expression profiling provided us with a complex view of the spatiotemporal changes that occur during chronological ageing of *S. cerevisiae* colonies passing through distinct acidic and alkali developmental phases and undergoing ammonia signaling-related U/L cell differentiation [6, 9, 13]. Major morphological and physiological differences between the U and L cell subpopulations become fully developed within alkali-phase colonies, and U and L cells are sharply demarcated within these colonies [6, 13]. However, new data provides evidence that some of the features typical of U and L subpopulations have already started to develop in earlier phases and that, while prominent expression changes exist in individual genes between day 6 and day 15, especially in upper cells, major DE functional gene categories are relatively preserved. Functional categorization thus shows which metabolic and other processes are important in each of the colony subpopulations, what the relationships between the particular cell types are, and what their similarities and differences are in relation to their age and position within the colony. However, more detailed analyses of individual genes combined with additional biochemical analyses will be needed to understand the dynamics of changes within particular functional groups and thus changes related to particular metabolic processes and functions of individual genes.

## 4. Materials and Methods

### 4.1. Strains and Cultivation

*S. cerevisiae* strain BY4742 (*MATa*, *his3*∆, *leu2*∆, *lys2*∆, and *ura3*∆) was obtained from the EUROSCARF collection. Yeast giant colonies (6 per plate) were grown at 28°C on GMA (1% yeast extract, 3% glycerol, 2% agar, 1% ethanol, 10 mM CaCl_2_, 0.05% glucose, and 0.002% uracil) with pH dye indicator bromocresol purple.

### 4.2. Separation of Cell Subpopulations and RNA Isolation

Individual cell layers were harvested from colonies by micromanipulation as described previously [17]. Three particular cell populations were separated from 6-day-old acidic-phase colonies (samples M6, U6, and L6) and from fully differentiated 15-day-old colonies in the alkali phase (samples M15, U15, and L15). Biomass of each cell sample was harvested from at least 60 colonies (day 6) or 24 colonies (day 15). Cells from 15-day-old colonies were stepwise separated into four smaller subpopulations called U1 (“U upper” cells), U2 (“U lower” cells), L1 (“L upper” cells), and L2 (“L lower” cells), that is, U and L cell layers were equally divided into two individual cell subpopulations. Biomass of each small subpopulation was harvested from at least 24 differentiated colonies. The RNA was isolated from three independent biological replicates. The purity of the cell fractions was controlled by Nomarski contrast microscopy. Total RNA was extracted using the hot-phenol extraction procedure as previously described [25]. To verify the quality and quantity of the RNA extracted, spectrophotometric and electrophoretic analyses were performed. The absorbance of the samples was analyzed at 230, 260, and 280 nm. The total RNA (20 *μ*g) from each replicate was DNase treated using a GenElute™ Mammalian Total RNA Miniprep Kit (Sigma-Aldrich).

### 4.3. cDNA Library Preparation and RNA Sequencing

Total RNA (4 *μ*g) from each replicate was spiked with 1 *μ*l of ERCC RNA Spike-In Control Mix 1 (Life Technologies) according to manufacturer's instructions. Ribosomal RNA was depleted from all samples using the Ribo-Zero Gold (yeast) rRNA Removal Kit (Illumina Inc., San Diego, CA) followed by purification using Agencourt RNAClean XP reagents (Beckman Coulter, Brea, CA). Efficient rRNA removal was confirmed using a 2100 Bioanalyzer system (Agilent Technologies, Santa Clara, CA). Strand-specific sequencing libraries were prepared from 50% of the depleted rRNA (equivalent to 2 *μ*g total RNA starting material) using a TruSeq Stranded Total RNA Library Prep Kit (Illumina Inc., San Diego, CA) with dual-indexed adapters, employing 15 PCR amplification cycles. A single pool of all samples was sequenced on two lanes of an Illumina HiSeq 3000 system (Illumina, San Diego, CA) with 150 bp paired-end reads, yielding a high-quality, high-coverage transcriptome library with ~12.6 to 23 million sequencing reads per replicate. 96–98% of reads were mapped to 10,409 coding and noncoding loci or to intergenic regions of the yeast genome. For all replicates, 52–72% of mapped reads were mapped to coding or noncoding loci, 16–33% to 5′ and 3′ UTRs, 9–15% to intergenic regions, and 0.3–1% to introns (Figure S1A). 54–82% of reads, mapping to genomic loci, mapped to coding genes and 18–45% to lncRNA (long noncoding RNA) loci (Figure S1B). Retention of strand information was confirmed by visual inspection using the Integrative Genomic Viewer [26].

### 4.4. Read Mapping, Counting, and Differential Expression Analysis

Reads were mapped to the SacCer3 reference genome and to a custom transcriptome (GTF file), incorporating the coding loci of *S. cerevisiae* build R64 (Ensembl release 76, [27]), ERCC spike-in transcript loci [28], and deduplicated lncRNA loci longer than 200 bp from previous studies [29–33]. Mapping was carried out using the STAR aligner [34]. Mapped reads were counted using “featureCounts” [35] from the Bioconductor RSubreads package v1.22.2. Read counts for technical replicates were added together to produce biological replicate counts. DESeq2 v1.12.3 [36] within R version 3.3.1 was used for differential expression analysis, and normalization/removal of unwanted variation (arising from technical effects) was carried out using RUVseq package v1.10.0 [37] from Bioconductor, using 36 spike-in transcripts, with interreplicate standard deviation/mean count ratios below 0.3, as negative controls.

### 4.5. Validation of RNA-seq Analysis by qPCR

qPCR was designed to verify the differences in gene expression of selected target genes identified by RNA-seq analysis. A 1 *μ*g sample of RNA (the same as that used for RNA-seq) was used to synthesize first-strand cDNA. The reverse transcription reactions were performed with Random Primer Mix (S1330S, NEB) using the SuperScript™ III Reverse Transcriptase (Invitrogen) following the manufacturer's instructions. The mRNA levels of target genes were quantified by real-time PCR analysis on LightCycler®480II (Roche) using SYBR® Select Master Mix (Applied Biosystems) with 2 *μ*l of template cDNA (5-fold dilution after RT reaction) in 10 *μ*l reaction volume according to the manufacturer's protocol. All reactions were run in triplicate and cycle threshold (*C*_T_) values for target genes were normalized to up to 5 housekeeping genes (*RPB2*, *TAF6*, *COG1*, *RAD52*, and *RDN25-1*) selected according to their expression profiles in cell populations. 15 genes with different expression in different cell populations were selected as targets. Primers designed for each gene are given in Table S1. The fold change in the expression of the target genes was calculated using the formula: 2^−ΔΔ*C*^_T_, where Δ*C*_T_ = average *C*_T_ of the target gene − average *C*_T_ of endogenous control (housekeeping gene), and ΔΔ*C*_T_ = Δ*C*_T_ of the target sample (cell population 1) − Δ*C*_T_ of the calibrator sample (cell population 2). The mean of normalized values was used for comparison with RNA-seq data.

### 4.6. Respiration Rate Measurement

The oxygen consumption of 2 mg of freshly isolated U6, M6, L6, U15, M15, and L15 wet cell biomass was determined at 30°C in 1 ml of water using a 782 oxygen meter with a 1 ml MT-200A cell (Strathkelvin Instruments) as described previously [7].

### 4.7. FC Enrichment/Reduction among DE Genes

Fisher's exact test was used to assay the significance of FC enrichment/underrepresentation among genes, up- or downregulated in a particular comparison and the Benjamini-Hochberg procedure used to control the false discovery rate. These steps were carried out using rcompanion package 1.10.1 [38]. A FC was deemed to be enriched/underrepresented among an upregulated gene dataset if the percentage of upregulated genes that were included in the FC was significantly higher/lower (*p*_adj_ < 0.05) than the percentage of genes in the yeast genome that belong to the same FC.

## Supplementary Materials

Supplementary Materials Figure S1: Read mapping. For each sample, the percentages of reads, mapping to different genomic features (A), and the percentages of locus-mapped reads, mapping to genes and to lncRNA (B), were calculated and plotted as bar charts. Table S1: List of the primers.
