# Supplementary material for: Transcriptome Remodeling of Differentiated Cells during Chronological Ageing of Yeast Colonies: New Insights into Metabolic Differentiation
Source: Oxid Med Cell Longev. 2018 Jan 11;2018:4932905. doi: 10.1155/2018/4932905 (PMC5821948; doi:10.1155/2018/4932905)
Supplement: Supplementary Materials — Figure S1: Read mapping. For each sample, the percentages of reads, mapping to different genomic features (A), and the percentages of locus-mapped reads, mapping to genes and to lncRNA (B), were calculated and plotted as bar charts. Table S1: List of the primers. [file 4932905.f1.pdf]

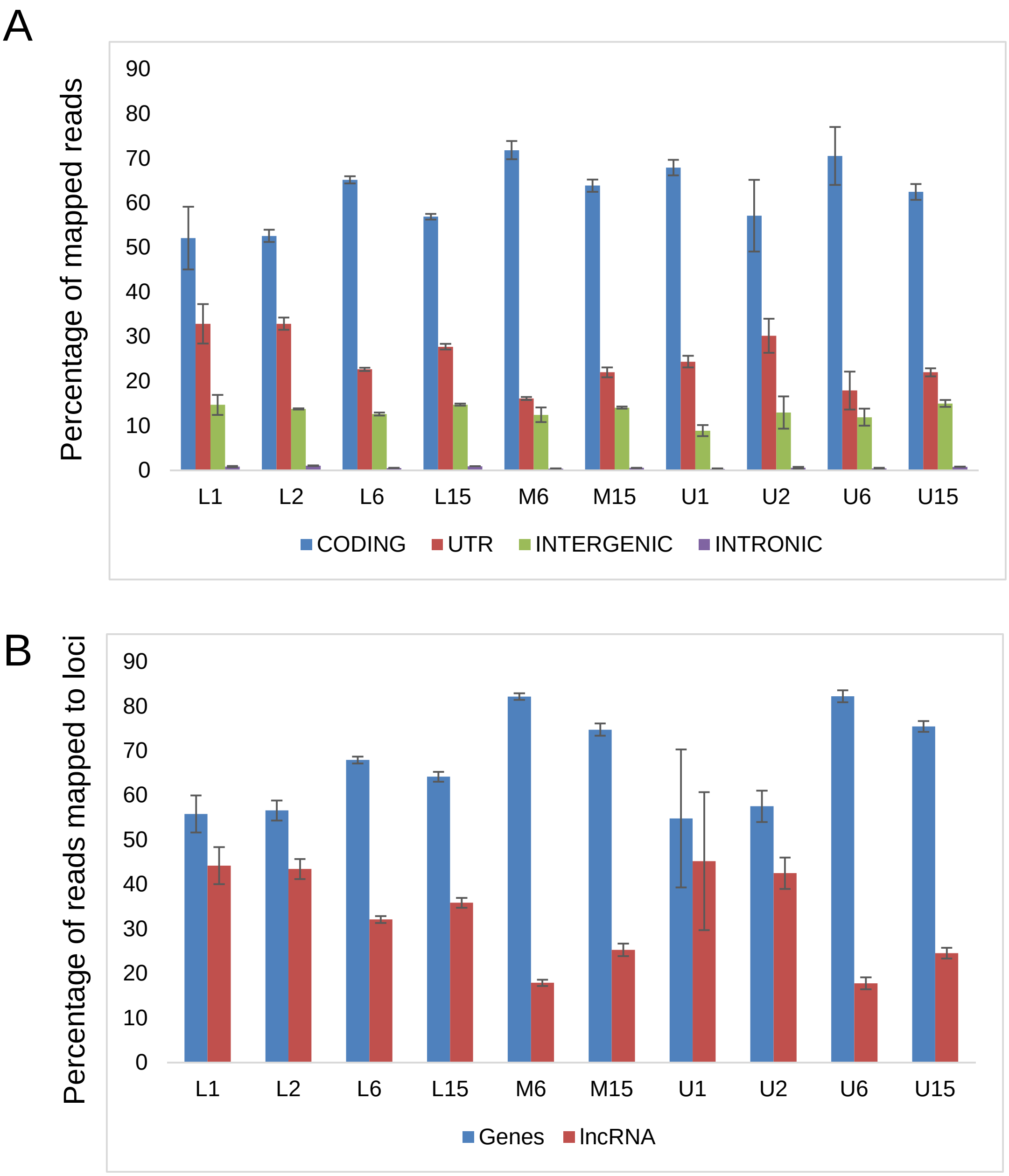


**Figure S1**

**Read mapping.** For each sample, the percentages of reads, mapping to different genomic features (A) and the percentages of locus-mapped reads, mapping to genes and to lncRNA (B), were calculated and plotted as bar charts.

## Table S1: List of the primers

| | |
|---|---|
| RDN25-1-int-for | GAGGAAAAGAAACCAACCG |
| RDN25-1-int2-rev | AGGCACTTTACAAAGAACCG |
| RPB2-int-for | GAAGACGATGAATCACTAGG |
| RPB2-int-rev | TCAGCATCAATGTATTCCAC |
| COG1-int-for | AGTTACTTCCACTACTCCG |
| COG1-int-rev | TGTCTTCATAGAGTCCACC |
| TAF6-int-for | ATTAGAGGTGCTATTGTCAC |
| TAF6-int-rev | CACTAAAGGTTTGACTTCAG |
| RAD52-int-for | AGAACAACAACAATACCCG |
| RAD52-int-rev | TACTGTTAGCCTCAGCAG |
| NCE102-int-for | CATCGGTTTAATCAGTTCG |
| NCE102-int2-rev | CCAATGGTTCAATGAAGTTG |
| VBA5-int-for | AGGAGATAGAAGGAGCATG |
| VBA5-int-rev | ACTGTAGCCTGTAACGAG |
| YDL218w-int-for | GAATGTACTTCTGTGCCG |
| YDL218w-int-rev | TACATGCTCTTCTATGACTG |
| MIG2-int-for | GATACACCAACCAATACCG |
| MIG2-int-rev | ATTGGCATAGGAGAAGTTG |
| CYC1-int-for | ATGTCTACAATGCCACACC |
| CYC1-int-rev | CTTCAACCCACCAAAGGC |
| CYC7-int-for | GTGTCAGCAGTGTCATAC |
| CYC7-int-rev | CTTCTTCAACCCGGCAAAC |
| HSP30-int-for | GGTTGGACTGGTGTTCAAG |
| HSP30-int-rev | CACGATTTGAATTAACAGCG |
| CTS1-int-for | ACTCAGCAGTCGTTGATG |
| CTS1-int-rev | TCCAACAAGTCACCAACAG |
| THI5-int-for | TACGGTATGAAGCCAGAAG |
| THI5-int-rev | AACGGTACAGAAGCAACAG |
| VHT1-int-for | CTGGACTTGGTTCACATTTG |
| VHT1-int-rev | CAGAAGTCCTGAAGTCACG |
| INO1-int-for | CCCACTGTTCAAGACTACG |
| INO1-int-rev | TTAACGCCTTCCTTAGTTTG |
| ZRT1-int-for | TGGTGGAAACGAGTACAATG |
| ZRT1-int-rev | CAATCGCACCATAAGCAG |
| ANS1-int-for | AGTAAGCAACTCCTCAGAC |
| ANS1-int-rev | CAACCACCCACATCATATAC |
| CWP1-int-for | CGGCTCGGATTTACAATAC |
| CWP1-int-rev | CATCCTCATTGACAACAGC |
| STL1-int-for | CTTGGTAGGAACACTAGACG |
| STL1-int-rev | GTATATGGCAGCGTTACAAC |
| PDR15-int2-for | ACTCACTCGGTTGTGTATG |
| PDR15-int-rev | CTGGTCTCCCAAGAACAAC |
